# Understanding the transmission dynamics of *Leishmania donovani* to provide robust evidence for interventions to eliminate visceral leishmaniasis in Bihar, India

**DOI:** 10.1186/s13071-016-1309-8

**Published:** 2016-01-27

**Authors:** Mary M. Cameron, Alvaro Acosta-Serrano, Caryn Bern, Marleen Boelaert, Margriet den Boer, Sakib Burza, Lloyd A. C. Chapman, Alexandra Chaskopoulou, Michael Coleman, Orin Courtenay, Simon Croft, Pradeep Das, Erin Dilger, Geraldine Foster, Rajesh Garlapati, Lee Haines, Angela Harris, Janet Hemingway, T. Déirdre Hollingsworth, Sarah Jervis, Graham Medley, Michael Miles, Mark Paine, Albert Picado, Richard Poché, Paul Ready, Matthew Rogers, Mark Rowland, Shyam Sundar, Sake J. de Vlas, David Weetman

**Affiliations:** London School of Hygiene and Tropical Medicine, Keppel Street, London, WC1E 7HT UK; Liverpool School of Tropical Medicine, Pembroke Place, Liverpool, L3 5QA UK; UCSF School of Medicine, 550 16th Street, San Francisco, 94158 CA USA; Institute of Tropical Medicine, Antwerp, Belgium; KalaCORE, London, Uk; University of Warwick, Gibbet Hill Campus, Coventry, CV4 7AL UK; European Biological Control Laboratory, USDA-ARS, Tsimiski 43 Street, Thessaloniki, 54623 Greece; Rajendra Memorial Research Institute of Medical Sciences, Patna, India; Genesis Laboratories, Inc., Wellington, CO 80549 USA; Bill and Melinda Gates Foundation, Seattle, USA; FIND, Campus Biotech, Chemin des Mines 9, 1202 Geneva, Switzerland; Institute of Medical Sciences, Banaras Hindu University, Varanasi, 221005 India; Department of Public Health, Erasmus MC, University Medical Center Rotterdam, Rotterdam, Netherlands

**Keywords:** Visceral leishmaniasis, Transmission, *Leishmania donovani*, Elimination, Control, Indian sub-continent, *Phlebotomus argentipes*

## Abstract

Visceral Leishmaniasis (VL) is a neglected vector-borne disease. In India, it is transmitted to humans by *Leishmania donovani*-infected *Phlebotomus argentipes* sand flies. In 2005, VL was targeted for elimination by the governments of India, Nepal and Bangladesh by 2015. The elimination strategy consists of rapid case detection, treatment of VL cases and vector control using indoor residual spraying (IRS). However, to achieve sustained elimination of VL, an appropriate post elimination surveillance programme should be designed, and crucial knowledge gaps in vector bionomics, human infection and transmission need to be addressed. This review examines the outstanding knowledge gaps, specifically in the context of Bihar State, India.

The knowledge gaps in vector bionomics that will be of immediate benefit to current control operations include better estimates of human biting rates and natural infection rates of *P. argentipes*, with *L. donovani*, and how these vary spatially, temporally and in response to IRS. The relative importance of indoor and outdoor transmission, and how *P. argentipes* disperse, are also unknown. With respect to human transmission it is important to use a range of diagnostic tools to distinguish individuals in endemic communities into those who: 1) are to going to progress to clinical VL, 2) are immune/refractory to infection and 3) have had past exposure to sand flies.

It is crucial to keep in mind that close to elimination, and post-elimination, VL cases will become infrequent, so it is vital to define what the surveillance programme should target and how it should be designed to prevent resurgence. Therefore, a better understanding of the transmission dynamics of VL, in particular of how rates of infection in humans and sand flies vary as functions of each other, is required to guide VL elimination efforts and ensure sustained elimination in the Indian subcontinent. By collecting contemporary entomological and human data in the same geographical locations, more precise epidemiological models can be produced. The suite of data collected can also be used to inform the national programme if supplementary vector control tools, in addition to IRS, are required to address the issues of people sleeping outside.

## Introduction

Visceral Leishmaniasis (VL), or kala-azar, is a neglected vector-borne disease. In India, it is transmitted to humans by *Leishmania donovani*-infected *Phlebotomus argentipes* sand fly females and typically affects the poorest of the poor [1]. It is endemic in the states of Bihar, Jharkhand, West Bengal and Uttar Pradesh, but Bihar contains more than 90 % of the cases [2]. In 2005, the governments of India, Nepal and Bangladesh, in collaboration with the World Health Organization (WHO), developed a strategic framework to eliminate VL as a public health problem by 2015. This was defined as reducing the annual VL incidence below 1/10,000 people at the block level [3]. The elimination strategy consists of rapid case detection, treatment of VL cases and vector control using indoor residual spraying (IRS). A toolkit was later produced by the three countries, in collaboration with TDR, to provide guidance for optimisation and monitoring and evaluation of the entomological intervention [4]. The toolkit recommended that the timing of spraying, and the number of spray rounds required, should take in to account the two annual density peaks of *P. argentipes*. The peaks occur around May-August and October-November in India [5, 6].

Bangladesh and Nepal opted to use pyrethroids for IRS, and India chose to continue to use DDT. However, the decision to use DDT over pyrethroids in India has been criticised by members of the scientific community, due its negative impact on the environment. In addition, DDT use is only sanctioned under the Stockholm convention as an insecticide of last resort when resistance to alternatives precludes their effective use. In India, concerns were raised regarding the development of DDT resistance in *P. argentipes* [7]. There is now compelling evidence, provided by a comprehensive quality assurance and insecticide resistance study, to suggest that DDT-based IRS in India is suboptimal and resistance of *P.argentipes* to DDT is widespread [8]. A further fear is that populations of *P. argentipes* may have changed their behaviour from being predominantly endophilic (resting indoors) to exophilic (resting outdoors) as a consequence of DDT-based IRS [9]. If this is true, and if many vectors are not resting indoors for a sufficient period of time to acquire a lethal dose of insecticide, then control may be compromised.

In 2015, the National Vector Borne Disease Control Programme (NVBDCP) of India introduced an alpha-cypermethrin-based IRS, in seven districts of Bihar State, with the view to rolling out pyrethroid-based IRS across Bihar if it proves to be more effective than DDT-based IRS. An ongoing research trial, performed by researchers at the Rajendra Memorial Research Institute of Medical Sciences, India, with the Liverpool School of Tropical Medicine, UK, will determine whether pyrethroid-based IRS is more effective in reducing densities of *P. argentipes* than DDT-based IRS when both are implemented according to best practice in two districts of Bihar, Muzaffarpur and Vaishali. Although the results of this trial will support evidenced-based IRS implementation by the NVBDCP, many questions concerning *P. argentipes* behaviour and VL case detection remain unanswered.

A systematic review of risk factor analyses for VL in South Asia, and its implications for elimination, was published over 5 years ago [10]. The review identified some fundamental evidence gaps that should be addressed in order to effectively promote VL elimination in South Asia. Since then, at least three position papers discussing the progress that has been made with existing interventions [11–13], and two systematic reviews, focusing on VL diagnostics [14] and VL transmission modelling [15] respectively, have been published. In the absence of an effective prophylactic vaccine, focus should be directed in the near future on further improving the interventions currently used in operational programmes supported by basic research to better understand the epidemiology of the disease.

To achieve sustained elimination of VL, an appropriate post elimination surveillance programme needs to be designed, and therefore crucial knowledge gaps in vector bionomics, human infection and transmission must be addressed. This will be of immediate benefit to current control operations as it will allow optimisation of vector control, case detection and management strategies. This review aims to identify the outstanding knowledge gaps, specifically in the context of Bihar State, India.

## Review

### The knowledge gaps in vector bionomics

It is rare to collect both entomological and human data in parallel during VL control programmes. One study, a cluster-randomized controlled trial [the KALANET project using long lasting insecticide treated nets (LLINS) for vector control] showed that a 25 % reduction in *P. argentipes* density/house [16] was not sufficient to result in any significant clinical impact, as measured by the risk of seroconversion over the 24 months of intervention, nor in reducing the risk of clinical VL [17]. In contrast, insecticide impregnation of existing bed-nets in an operational research project reduced VL by 66.5 % in Bangladesh [18]. One hypothesis proposed to account for the difference in outcomes between the two different trials was that historical use of IRS in India and Nepal, compared with Bangladesh, may have led to behavioural or physiological adaptation/habituation in *P. argentipes* [19]. Another possibility may relate to differences between sleeping preferences of humans. Although it has been reported that bed nets were used in all seasons by 50.6 % of Indian household [20], another study conducted in the Saran district of Bihar reported that 95 % of households sleep outside [2]. Recent data suggests that over 88 % of VL patients sleep outside for 5-8 months (Poché, unpublished data) coinciding with the May-August peak in *P. argentipes* populations. The significance of sleeping outdoors for VL transmission warrants further investigation.

After elimination targets are reached it will be necessary to establish a rapid detection, treatment and response system to identify transmission hot-spots and avoid outbreaks or resurgence in transmission. A switch from IRS campaigns to more precise, targeted or sustainable methods of vector control or individual protection may prove more appropriate in these circumstances. The exact format or mix of outdoor and indoor vector control that lends itself to such a rapid response system would need to be determined and evaluated prior to state-wide implementation. In parallel, data on the dispersal of *P. argentipes*, which is poorly understood, must be collected to advise operational teams regarding the focal area that needs to be sprayed around clusters or houses of active VL cases, if this is the method of choice in the new response mode.

Few attempts have been made to produce epidemiological models for VL transmission and control in the Indian subcontinent [15, 19, 21] but, as recognised by these authors, models must be improved and refined by inclusion of robust and recent data collected in the geographical region of interest. For instance, much of the data used to provide model parameters for *P. argentipes* were either collected in Nepal, on natural infection rates with *L. donovani* (e.g. [22]), or, with respect to feeding cycle duration, over 20 years ago in India [23]. Consequently, not only do the models still lack parameterisation [15] but they may not be able to accurately simulate current VL transmission. Therefore, contemporary data from Bihar concerning the density of *P. argentipes* (in absolute numbers or per human), *P. argentipes* life expectancy, infection prevalence, blood-feeding cycle duration and sojourn time of *P. argentipes* in the latent stage are required. One factor affecting the infection rate of *P. argentipes* with *L. donovani* is the sand fly’s biting rate, but there are a limited number of studies that have standardised sampling methods for estimating biting rates sufficiently to usefully complement epidemiological models integrating risk analyses and basic reproduction number (*R*_0_) [24, 25].

It is ethically unacceptable to use humans as bait for estimating biting rates, but capturing sand flies and molecularly analysing their blood meals can provide indirect estimates. The most common methods for capturing *P. argentipes* are aspirator catches and/or CDC miniature light traps, which are usually performed in human dwellings or in cattle sheds, but specific details concerning trap placement, and measures taken to standardise collections, are often missing from surveys, making comparisons between them difficult. Furthermore, collections of blood-fed females are often limited, which adds to the potential bias arising through sampling procedures and can lead to data misinterpretation. For example, it is not surprising that most *P. argentipes* collected in cattle sheds ingested bovine blood and sand flies collected in houses contained mainly human blood [10].

A more recent study performed in three villages in Bihar extended collections to a wider range of sampling sites and over a longer sampling period [26]. In addition to houses, cattle sheds and mixed dwellings, CDC light traps were placed in other potential resting sites such as chicken sheds and vegetation (banana, palm, bamboo etc.). A total of 288 blood meals were PCR identified in *P. argentipes* using cytochrome b: humans were the dominant source in the samples collected, followed by cattle, and mixed human/cattle meals in blood-fed sand flies were quite common. However, this method cannot discriminate between blood meals from a single human or mixed meals from different humans. The potential for the same sand fly to feed on more than one human host, which may be influenced by infection of the vector [27], could have important epidemiological implications and should be explored further. The study also found that vegetation contained the second highest proportion of blood-fed sand flies (26 %), after combined dwellings (31 %), then cow sheds (24 %), houses (15 %) and poultry sheds (4 %) [26]. These findings, together with the fact that an average of 30 *P. argentipes* were collected per trap night in the canopies of Palmyra palm trees at heights of 18 metres above the ground in Bihar [28], have shed new light on the exophilic behaviour of *P. argentipes*. The extent of exophily has implications for control. If exophily is more important than previously recognised, then control tools targeting exophilic behaviour may be required to supplement IRS.

When using human and cattle baits, *P. argentipes* collections are several-fold higher on cattle than humans over the same period of exposure, and it was suggested that this may be a consequence of host density or biomass on sand fly blood-feeding behaviour [10], but may also reflect the heterogeneous distribution of sand flies between available hosts resulting from aggregations (leks), which *P. argentipes* are known to form [29]. One field study, that attempted to take host availability into consideration, was performed in Bihar with the main objective to determine whether host preference of *P. argentipes* changed as a result of DDT-based IRS used in the VL elimination programme [9]. Additional studies of this nature are required, especially given that disruption of recruitment to aggregation sites, following the loss of conspecific cues due to IRS induced mortality, may amplify any insecticide-induced repellence or changes in host preference and divert sand flies towards other sites such as non-covered households/animal sheds. Such behaviour has been experimentally observed in the heterogeneous spraying of chicken sheds in the control of another lekking sand fly species, *Lutzomyia longipalpis*, in Brazil [30].

The first study to examine natural infection rates of *P. argentipes* with *L. donovani* in Bihar was published relatively recently [31]: a total of 14,585 sand flies were collected using CDC miniature light traps and mouth aspirators from nearly 900 houses selected from 50 villages in the Muzaffarpur district. Of these, 449 were *P. argentipes* females which were divided into 132 pools for molecular detection of the 18S rRNA gene using PCR. The overall prevalence of infection for *L. donovani* in *P. argentipes* was estimated to be between 4.90-17.37 % across the region. These rates are surprisingly high and were probably because the use of pooled samples in the analysis was not considered. More recent and reliable estimates, using individual sand flies, showed that there may be seasonal changes in natural infection rates of *P. argentipes* with *L. donovani*: 1.0 % (4/384) in summer, 0.9 % (5/591) in the rainy season, and 2.8 % (12/422) in winter [32]. Such estimates should be repeated, ideally over time, and obtained for parous females only [24, 25].

Although there are molecular tools available to determine natural infection rates, a measure of infectiousness (whether *L. donovani* has developed to the infective metacyclic stage for transmission to humans) is particularly relevant to epidemiological models. Quantification of the proportion of sand flies that are infective, together with the human blood index (HBI), is required to calculate the entomological infection rates (EIR) for *L. donovani* in *P. argentipes*. This information is crucial to determine the intensity of transmission in a particular area, to accurately evaluate the impact of control measures on transmission. Key to this is the development of suitable molecular tools for the deployment of a metacyclic-specific qPCR optimised for use in *L. donovani*-infected *P. argentipes*.

In summary, in order to inform surveillance and improve control activities of *P. argentipes* in Bihar, there are several research questions relating to sand fly population dynamics and behaviour that remain outstanding (see Table 1).

**Table 1:** Research questions relating to sand fly population dynamics and behaviour to inform surveillance and improve control of *P. argentipes* in Bihar

• What are the blood-feeding preferences of *P. argentipes*, and how do they vary spatially and temporally (diurnally and seasonally), in response to IRS (diversion)?	
• Do natural infection rates of *P. argentipes* with *L. donovani* vary spatially, temporally and in response to IRS (diversion)?	
• How far does *P. argentipes* disperse within villages?	
• What is the EIR for *L. donovani* in *P. argentipes*?	
• What is the relative importance of indoor and outdoor transmission in relation to IRS?	
• Is IRS selecting for resistance and, if so, what are the mechanisms and at what rate is it emerging in the field?	
• If IRS as the stand-alone vector control tool does not stop transmission, what are the appropriate available tools to control residual transmission?	

### The knowledge gaps in human infection and transmission

*Leishmania donovani* infection in humans leads to clinical disease in only a fraction of all those infected, and ultimately to death if the symptomatic patient is not treated. Many people living in endemic villages are positive for one or more of several infection markers, either for antibodies (DAT, rK39), parasite DNA (PCR) or cellular immunity markers, but the exact meaning of those test results is not clear because these people are asymptomatic for VL infection at the time of testing [33]. The lack of simple and validated markers to determine infection/exposure status in humans makes it difficult to demonstrate the effect of vector control interventions and their impact at population level. More generally, the endgame of VL elimination demands a better understanding of the role of those “asymptomatics” in transmission and much stronger epidemiological surveillance [13]. There is a consensus by the Regional Technical Advisory Group on VL elimination that the current health information system in the region needs to be strengthened in this respect [3]. Population-based long term follow-up data obtained from sentinel surveillance sites could help to underpin declining VL trends observed in routine data reporting systems. However, much better validated serosurveillance markers are needed, especially markers for past exposure to infection (or to sand fly bites), to exploit such serosurvey data optimally.

It has been shown that individuals with high antibody titres have a substantially higher risk of progressing to VL disease [34] and they are thought to also be the most infectious to sand flies, but this still needs to be confirmed. To demonstrate recent infection, paired samples to show conversion in antibody tests or in PCR are usually performed, but there is little agreement between the two methods. The Leishmanin Skin Test (LST) usually assesses past exposure, but this is not accepted in India as there is no source of Good Manufacturing Practices (GMP)-produced antigen. A possible alternative for the LST is the Interferon Gamma Release Assay (IGRA), but despite evidence that it correlates with past exposure it has not yet been applied at population level. The development and production of leishmanin antigen under GMP to enable LST surveys in the future should be encouraged.

Sand fly saliva antibody detection is a surrogate marker for bites that could be used for measuring exposure to sand flies, a concept that has proven useful in other vector-borne diseases such as malaria to monitor the impact of vector control efforts. Beyond determining intervention efficacy, anti-saliva antibody assays could be incorporated into surveillance protocols, particularly after the rollout of a vector control intervention, to monitor when the sand fly population begins to rebound and interventions need re-application. With regards to expanding knowledge on sand fly bionomics, bite exposure can be a useful epidemiological tool to monitor the spatial distribution of sand flies in a foci/region. This could then be used to inform the vector control teams where to spray, which would inevitably help decrease sand fly biting and lower the risk of contracting VL. Finally, monitoring anti-saliva antibodies in sera can also help pinpoint human behaviours that increase exposure to sand fly biting, which could then help direct future educational and vector control campaigns. Although proof of concept for the validity of this assay was provided during the KALANET trial [35] there is a need to optimize and evaluate an assay with recombinant *P. argentipes* salivary antigen or antigens [36].

In summary, it is important to optimise and then deploy the aforementioned prototype assays in clinical and community studies to improve the understanding of transmission to humans. These results will then be used to inform future serosurveillance strategies, and improve active case detection in foci of disease [37], in order to evaluate the impact and sustainability of elimination. More specifically, it is important to distinguish individuals in endemic communities into those who: 1) are to going to progress to clinical VL, 2) are immune/refractory to infection and 3) have had past exposure to sand flies.

Today, there is a set of potential markers of infection available that can be assessed in a single blood (high titre-rK39 ELISA; high-titre-DAT; IgG1 ; qPCR) or urine sample (antigen detection assays) which may be able to identify those who are going to progress to clinical VL [34, 38]. Apart from their relevance for public health use in epidemiological surveillance, these markers, if validated, could have clinical benefit as rapid treatment may improve the patients’ clinical outcome. If progressors are equated to being the most infectious individuals, then these assays will be relevant for targeted vector control measures in their immediate surroundings to prevent further transmission. Validated infection/infectiousness markers will also facilitate xenodiagnostic studies which are currently struggling to define the role of potentially infectious asymptomatics in disease transmission.

Secondly, for surveillance of VL elimination, it will be important to identify individuals who are immune/refractory to infection, e.g. through the use of a GMP-compliant LST or the IGRA marker as a measure of cellular immunity, in order to monitor changes in population susceptibility over long timescales (e.g. post-elimination surveillance) and, with the added advantage of the IGRA, to inform future vaccine studies. To evaluate heterogeneity in exposure risk and to monitor effectiveness of vector control interventions, individuals who have had past exposure to sand flies should also be identified and this can be achieved using sand fly saliva antibody detection with recombinant antigen.

However, in order to optimize and validate existing prototype markers for detection of past exposure, putatively highly infectious individuals and progressors to active VL cases, several research questions need to be addressed (see Table 2).

**Table 2:** Research questions to address human infection and transmission to validate and optimize existing prototype markers for detection of past exposure, putatively highly infectious individuals and progressors to active VL

• What is the value of new prototype point of care (POC) tests (IgG1 RDT; urinary antigen detection) in comparison with existing quantitative antibody tests (and qPCR) to detect those healthy but infected individuals who are likely to progress to clinical VL (progressors)?	
• What is the value of IGRA as an alternative to LST (which is not currently allowed for use in India) for documenting the presence of cellular immunity (exposed but not susceptible) at population level?	
• What is the validity of sand fly saliva antibody detection for detecting past exposure?	
• Are progressors to VL a significant source of infection to sand flies as compared to non-progressors?	

Such markers may also be used to assess the impact of Indian VL elimination efforts on transmission and to address whether there are changes in age-specific prevalence patterns compared with historical sero-survey data [39].

### Addressing knowledge gaps by modelling VL transmission to ensure sustained elimination

Better understanding of the transmission dynamics of VL, in particular of how rates of infection in humans and sand flies vary as functions of each other, is required to guide VL elimination efforts and ensure sustained elimination in the Indian subcontinent. A number of key questions must be addressed (see Table 3), including determining the spatial and temporal patterns of transmission and the extent to which asymptomatic individuals contribute to transmission. Currently the latter issue is unclear. There is evidence that new VL cases are much more likely to appear in the same households as, or within a 50 m radius of, previous VL cases [40, 41]. However, these findings can also be partly explained by household-related factors that may expedite progression from infection to VL disease, such as poverty and poor nutritional status. Moreover, the findings of isolated (not travel-related) VL cases in very low endemicity settings [42, 43] indicates that transmission also occurs from asymptomatic individuals.

**Table 3:** Research questions to address knowledge gaps in VL transmission to optimise control strategies and ensure sustained elimination

• What are the spatial and temporal patterns of VL transmission, and how spatially heterogeneous are they?	
• Are VL cases a good indicator for infection prevalence?	
• How big is the pool of asymptomatics and how much do they contribute to transmission?	
• What is the optimal response strategy to new cases, e.g. in what radius around new cases should vector control be performed and for how long?	
• What is the right surveillance strategy to ensure sustained elimination? What markers should be used and which age groups should be tested?	

Mathematical and statistical modelling can assist in answering these questions, but are currently hampered by a lack of robust data with which to estimate key parameters. Despite this, previous models of VL have provided valuable insight. For example, a multivariate meteorological model developed by Picado *et al.*, 2010, where *P. argentipes* density was positively associated with temperature but negatively associated with rainfall, was able to explain 57 % of the monthly *P. argentipes* abundance in India and Nepal [5]. With further refinement, it may be possible to predict VL epidemics on the Indian subcontinent more accurately by monitoring simple meteorological variables (i.e., temperature and rainfall). Spatial patterns of *P. argentipes* and risk of VL have been studied with respect to proximity to water bodies and other environmental variables in the Vaishali district of Bihar [44, 45]. However, such models cannot be used to predict the impact of control and the prospects of elimination.

A dynamic transmission model investigating the effect of different interventions against *P. argentipes* in the Indian subcontinent used simulations to predict that elimination of VL is possible if the density of *P. argentipes* can be reduced by 67 %, e.g. using IRS or LLINs [19]. However, although IRS was shown to reduce *P. argentipes* density by 72 % in one study [46], the modellers advised that IRS should be combined with LLINs and more effective environmental vector management to prevent re-emergence of VL after local extinction [19]. Recent explorations with variants of the same model demonstrated that elimination depends strongly on the pre-control endemicity level and the quality of IRS [47].

Although some modelling studies have attempted to estimate key parameters in the transmission of VL, such as the infectiousness of asymptomatic individuals to sand flies and the duration of different disease stages [21, 48], there is still much uncertainty about the natural history of the disease. For instance, estimates suggest that the incubation period for VL lasts 2-6 months on average, but is highly variable [41, 48, 49]. This potentially has a significant impact on the transmission dynamics between humans and sand flies, and further studies are required to estimate the duration and variability of the incubation period across different endemicity settings. Along with better estimates of the contribution of asymptomatic individuals to transmission and sand fly life cycle parameters, this would help to give better estimates of the length of time for which IRS should be performed in areas where new VL cases appear. It is also not known to what extent successfully treated VL patients that develop PKDL (post kala-azar dermal leishmaniasis) contribute to transmission. Whilst this occurs for only around 5 % of the cases in India [50], PKDL can last for several years and may impede elimination and necessitate longer intervention programmes.

To improve our understanding of VL transmission dynamics and how the various components involved in transmission change in response to different interventions, studies are required to gather detailed sand fly data (as described in Section 1) alongside individual-level human data (with geo-referenced records of VL case history and serology measurements as described in Section 2) in a number of different locations (both with and without interventions) over at least a full transmission season. Furthermore, longitudinal epidemiological and entomological observations during interventions are essential.

It is important to keep in mind that close to elimination, and post-elimination, VL cases will become infrequent, so it is vital to define what the surveillance programme should target and how it should be designed to prevent resurgence. Previous studies show that incidence of VL varies with age, and that older children and young adults (5-15 years of age) are at higher risk of asymptomatic infection and developing VL [33, 41, 49]. This indicates that epidemiological surveys must be carried out across all age groups and that there may be a need for increased sero-surveillance of children to observe reduced transmission.

## Conclusions

Molecular tools enable the collection of accurate estimates of HBI in *P. argentipes*. The refinement of a metacyclic-specific qPCR assay to identify *L. donovani* in *P. argentipes* would enable quantification of EIR for the first time. Likewise, a set of prototype infection/exposure markers is currently available for which proof of concept and pilot laboratory data are already available and, if proven robust in larger-scale field evaluation, these markers will prove invaluable for future intervention trials of novel vector control methods and will provide essential population-based information on the long-term impact of interventions to inform a successful endgame for VL elimination.

A longitudinal study using these tools, in a representative number of intervention and control villages in different endemic districts is needed to determine whether pyrethroid-based IRS, when implemented by the NVBDCP, can reduce *P. argentipes* population densities to the critical level required to interrupt disease transmission. In addition to acquiring data to determine whether densities of *P. argentipes* females are lowered following intervention, precise entomological indices for *P. argentipes* (HBI and EIR) should be calculated to improve epidemiological models of VL. In parallel, improved diagnostic tools can be used to measure clinical outcomes when the number of active cases is very low.

By collecting contemporary entomological and human data in the same geographical locations and over a 2-year period, more precise epidemiological models can be produced, which are essential to inform the NVBDCP during the VL elimination endgame. The refined models will have the following applications: 1) predicting the reduction in disease that can be achieved using different intervention scenarios, 2) identifying hotspots for transmission and control, and 3) determining whether xenomonitoring of *P.argentipes* can be used in routine surveillance programmes, with an appropriate rapid response, to prevent VL transmission.

In addition, the suite of data collected can be used to inform the NVBDCP if supplementary control tools, in addition to IRS, are required to address the issues of people sleeping outside during months when sand fly densities are at their highest. Also, knowing whether the proportion of *P. argentipes* becoming exophilic, as a consequence of IRS, is increasing would be invaluable. There are a limited number of vector control tools available to roll out in disease control programmes that specifically target exophilic sand flies. One promising approach has been to treat cattle with systemic insecticides [51] and another is the targeted use of insecticides on localised vegetation or for the development of optimised attractive toxic sugar baits sand flies [52]. Should the refined models indicate that exophilic transmission impedes success at reaching the VL elimination target in endemic areas, further basic research will be required to develop additional tools to tackle this problem.

## Abbreviations

(R_0_): Basic Reproduction Number
CDC: Centers For Disease Control and Prevention
DAT: Direct Agglutination Test
DDT: Dichlorodiphenyltrichloroethane
EIR: Entomological Inoculation Rate
ELISA: Enzyme-Linked Immunosorbent Assay
IGRA: Interferon Gamma Release Assay
IRS: Indoor Residual Spraying
GMP: Good Manufacturing Practices
HBI: Human Blood Index
LLINs: Long Lasting Insecticidal-Treated Nets.
LST: Leishmanin Skin Test
PCR: Polymerase Chain Reaction
TDR: The WHO Special Programme for Research and Training in Tropical Diseases
VL: Visceral Leishmaniasis
WHO: World Health Organisation
